# A Simulation Methodology for Analyzing the Energy-Absorption Capabilities of Nanofluidic-System-Filled Tube under Split Hopkinson Pressure Bar Experiment

**DOI:** 10.3390/ma15197030

**Published:** 2022-10-10

**Authors:** Shuming Zhang, Ziqian Zhu, Shuaijun Li, Fei Yu, Chunping Tian, Lu Yao

**Affiliations:** 1 Wuhan Secondary Ship Design & Research Institute, Wuhan 430064, China; 2 China Ship Development & Design Center, Wuhan 430060, China

**Keywords:** energy absorption capabilities, nanofluidic system, split Hopkinson pressure bar, filled tube, simulation methodology

## Abstract

The energy-absorption mechanism of nanofluidic systems is being investigated under dynamic cases, represented by the split Hopkinson pressure bar experiment. However, the cost of this cannot be ignored. Therefore, numerical simulation is playing an increasingly important role in optimizing the split Hopkinson pressure bar experimental technology and analyzing its accuracy. In this paper, a three-dimensional finite element simulation model of the split Hopkinson pressure bar experimental devices was proposed to analyze the energy-absorption capabilities of nanofluidic-system-filled tubes. The reliability of this methodology was discussed in terms of model construction, model validation and potential application, indicating the simulation methodology is applicable to further investigation and can provide a reference for engineering practice. The simulation results showed that the infiltration pressure and the mass ratio of solid to liquid determine the post-buckling compression stress and the effective compression stroke, respectively.

## 1. Introduction

During recent years, when studying the nanofluidic behavior of liquid molecules entering and exiting the pores on a nanoporous material’s surface, some scholars have discovered that the thermodynamic system composed of lyophobic nanopores and a non-wetting liquid environment has the characteristics of absorbing, converting, accumulating, or dissipating energy [1,2,3,4].

**Figure 1:**
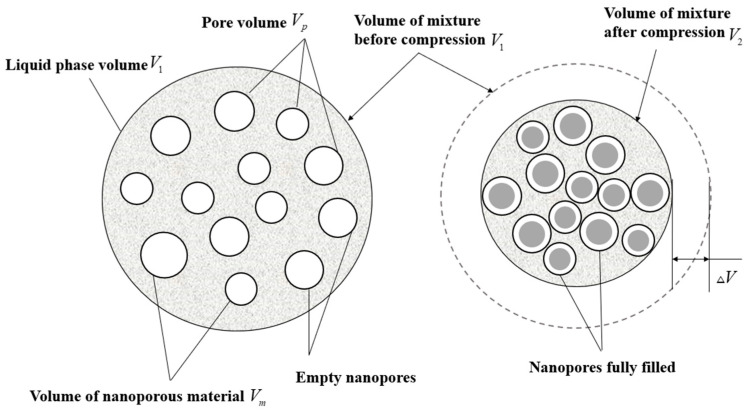
Schematic diagram of a nanofluidic system.

This system is named a ‘nanofluidic’ system, and is composed of a nanoporous material and liquid. Similar to a hydraulic buffer, the basic working principle of a nanofluidic system can be described as: under normal pressure, liquid molecules cannot spontaneously infiltrate into the hydrophobic nanopores, but they can be pushed to overcome the capillary repulsion force and infiltrate into the pore when the external pressure is stronger than a certain critical value, called the infiltrate pressure (Pcr) [5], during which part of the mechanical energy is converted into solid/liquid interface energy for storage [6,7]. When the external pressure disappears, the liquid molecules in the nanopores have a higher energy than those in the external environment, and they tend to seep out of the nanopores under the effect of the potential energy gradient [8] (see Figure 1). The friction between the liquid molecules and the pore will dissipate the energy regardless of infiltration or exudation. Therefore, a nanofluidic system can be regarded as a hydraulic buffer with damping holes. In fact, nanoporous particles have an amazing specific surface area (10^2^–10^3^ m^2^/g) [9,10], which is millions of times larger than ordinary hydraulic buffers. Therefore, the energy-absorption potential of systems involving nanofluids far exceeds that of any known material or structure.

As it is designed to protect against impact loads, the response of the system under dynamic impact provides useful references for application design. However, there are few experimental reports in this area, mainly including the split Hopkinson pressure bar experiment with high strain rate, and the drop-weight experiment with a medium and low strain rate. Surani et al. [11] used a nanoporous material–deionized water system made of Fluka 100-C8 silicone gel to carry out the split Hopkinson pressure bar experiment, and draw a conclusion that the energy-absorption density of the system is nearly three times that of quasi-static compression. Xu et al. [12] used silicon nanotubes with an aperture of 2.17 nm to conduct experiments with a lower impact rate (5 m/s), and also found that the energy absorption of the system under dynamic impact was much higher than that of the quasi-static compression experiment. Sun et al. [13] analyzed an energy-absorption system composed of β zeolite and water by using the drop-weight experiment with an impact rate of 1.98 m/s and an initial strain rate of 27.5 s^−1^. The results showed that the system can weaken 42.82% of the peak impact load and prolong the load duration by 79.16% compared to a control group of pure water. Li et al. [14] conducted a series of quasi-static and dynamic tests on different fluid-like liquid nanofoam filled tubes, and found the energy-absorption capacity showed 54% increase under dynamic ones, which is due to the energy dissipation at lower strain rate and energy capture at higher strain rate.

Despite the aforementioned experimental efforts of nanofluid materials, the general mechanisms of nanofluid systems under dynamic impact remain unclear, which hinders potential applications in engineering flexible, energy-absorbing materials. Such mechanisms can potentially be clarified using simulation; to this end, we explored a model system of filled tubes in a split Hopkinson pressure bar experiment using finite element simulations. The pressure bars and specimen were simulated according to the experiment. It offers a convenient method to explore the coupling between energy-absorption capabilities and the material characters of the nanofluidic-system-filled tube, which may provide important insight for the development and optimization of thermally responsive nanofluidic devices.

The main contents include the following aspects:

1. Establish a finite element model of the split Hopkinson pressure bar experimental device.
2. Establish a simulation method for a nanofluid system. Provide calculation support for the analysis of the split Hopkinson pressure bar experimental technology.
3. Determine the stress–strain curve of the specimen according to the parameters obtained by the Hopkinson bar simulation, which is used to calculate the energy-absorption capabilities.
4. Analyze the simulation results and discover factors that affect the energy-absorption capabilities of the nanofluidic system.

## 2. Split Hopkinson Pressure Bar Experiment Technique

### 2.1. Experimental Devices

**Figure 2:**
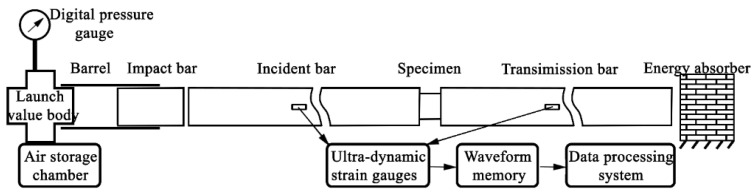
Split Hopkinson pressure bar experimental devices.

The split Hopkinson pressure bar device and data-processing system includes: pressure bars, a speed-measurement system, ultra-dynamic strain gauges, a transient waveform acquisition storage system, and a data-processing system, as shown in Figure 2. The contact surfaces of the pressure bars and the specimen were processed smoothly and kept parallel. The impact velocity was measured by a speedometer, and the strain pulses recorded by the strain gauges on the incident bar and the transmission bar are amplified by the super dynamic strain gauge and then collected and stored by the transient waveform memory. 

The nanofluidic-system-filled tube was investigated by Sun et al. [15] as shown in Figure 2. The tube wall was made by 06Cr19Ni10 stainless steel. The outer diameter of the tube was 6.35 mm, its thickness was 0.25 mm, and the height of the tube on the *y*-axis was the same as the outer diameter. The length of incident bar and transmission bar were 1778 mm and 1524 mm, respectively, and their diameters were both 127 mm. The material of bars was all stainless steel with a Young’s modulus of 1.97 × 10^11^ Pa, a density of 7.75 × 10^3^ kg/m^3^, and a longitudinal wave velocity of 5.04 × 10^3^ m/s.

### 2.2. Experimental Principles

The split Hopkinson pressure bar experimental technique is based on the following two basic assumptions: (1) one-dimensional stress of the elastic strut; (2) homogenization of the internal stress of the specimen.

A compression stress pulse *ε*_i_ is generated in the incident bar when the impact bar hits the incident bar axially. The sample is deformed at a high speed under the action of the pulse, and at the same time, a pulse *ε*_r_ is reflected back into the incident bar and a pulse *ε*_t_ is transmitted through the sample into the transmission bar. The strain gauges on the surface of the bars measure the signals, respectively. Based on the sample uniformity assumption, $\varepsilon_{i}+\varepsilon_{r}=\varepsilon_{t}$, the formula below (two-wave method) can be derived:

(1) $${\dot{\varepsilon }}_{s}=\frac{d\varepsilon_{s}}{dt}=-\frac{2l_{0}}{C_{0}}\varepsilon_{i}$$

(2) $$\varepsilon_{s}=\int {\dot{\varepsilon }}_{s}=-\frac{2l_{0}}{C_{0}}\int \varepsilon_{i}$$

(3) $$\sigma_{s}=\frac{EA_{0}}{A_{s}}\varepsilon_{t}$$

where *E*, *C*_0_, and *A*_0_ are the elastic modulus, wave velocity, and cross-sectional area of the compression bars, and *A_s_* and *l*_0_ are the cross-sectional area and thickness of the specimen, respectively. *ε*_s_ and *σ*_s_ refer to the stress and strain of the specimen.

Peak stress *σ*_max_, energy-absorption density (*EAD*), and crush-force efficiency (*CFE*) are introduced in order to quantify the energy-absorption capabilities. Peak stress refers to the maximum stress generated during the destruction of the component. The energy-absorption ratio refers to the energy value that a unit mass of material can absorb after being damaged, which can be simplified by obtaining the nominal stress–strain curve of the material/device, and can be expressed as follows:

(4) $$EAD=\int_{0}^{\varepsilon_{\max}}\sigma_{s}\left(\varepsilon_{s}\right)\;d\varepsilon_{s}$$

Among these, *ε*_max_ is the length of the failure section. *EAD* can be calculated by dividing the hysteresis area under the load-displacement curve by the total mass of the crushed material.

*CFE* emphasizes the flatness of the material/device during the failure process, which can be derived as follows:

(5) $$CFE=\frac{\sigma_{\mathrm{average}}}{\sigma_{\max}}=\frac{\int_{0}^{\varepsilon_{\max}}\sigma_{s}\left(\varepsilon_{s}\right)\;d\varepsilon_{s}}{\sigma_{\max}\varepsilon_{\max}}$$

Formula (6) can be obtained by combining Formulas (3) and (4) and substituting Formula (1) into the combination:

(6) $$EAD=\frac{EA_{0}}{A_{s}}\int_{0}^{t_{\max}}\varepsilon_{t}(t)\;d\varepsilon_{s}(t)$$

The strain–time curves of the bars (*ε*_t_ − t, *ε*_i_ − t) can be drawn by field output of a typical element in Abaqus. However, it is worth noting that the strain–time (*ε*_i_ − t) curve of the specimen can also be easily obtained through the displacement curves of the nodes near the specimen of pressure bars; therefore, Equation (6) does not need further simplifying.

## 3. Simulations and Discussions

### 3.1. Modeling of Split Hopkinson Pressure Bar Devices

The finite volume method is common in solving fluid problems; however, as the mechanical response of the energy-absorbing structure was considered, the fluid–structure interaction effect needs to be addressed first, if the finite volume method is used in our simulation. The mechanism of this effect under a high strain rate is complex, and the boundary conditions often need to be simplified. The simulation accuracy is very poor under the highly nonlinear condition, which does not meet the needs of the simulation in this paper. Therefore, finite element analysis was adopted.

An empty tube in a split Hopkinson pressure bar experiment was adopted to model first in order to verify the simulated experimental devices according to the experiment conducted [15]. The model consisted of an incident bar, transmission bar, and the empty tube. The geometric parameter settings of the bars and the empty tube were obtained from the experiment. The material property was assigned to incident bar and transmission bar. The model was uncomplicated; therefore, automatic grid generation was adopted by adding tight seeds. The mesh generation was performed with a reduced order integration (the number of Gaussian integration points selected is less than the integration points required for accurate integration) and S4R elements (four-node quadrilateral finite membrane strain linear shell element), and the Simpson integral count was set to five. 

The material of the tube wall was 06Cr19Ni10 stainless steel, the plastic behavior of which was investigated and discussed by Cook and Johnson [16]. The expression is as follows:

(7) $$\sigma_{y}=[A+B{\left(\varepsilon_{p}^{\mathrm{eq}}\right)}^{n}]\cdot [1+c\ln(\frac{\varepsilon_{p}^{\mathrm{eq}}}{{\dot{\varepsilon }}_{0}})]\cdot [1-{\left(\frac{T-T_{\mathrm{room}}}{T_{\mathrm{melt}}-T_{\mathrm{room}}}\right)}^{m}]$$

The parameter settings of the 304 stainless steel constitutive model were as follows: The density of the 06Cr19Ni10 stainless steel was 7900 kg/m^3^. Young’s modulus and Poisson’s ratio can be obtained by referring to relevant manuals, and were 200 GPA and 0.3, respectively. *n* and *m* were equal to 0.65 and 1, respectively. *T*_room_ was the ambient temperature of 293 K, *T*_melt_ was the melting point of 1673 K. ${\dot{\varepsilon }}_{0}$ and *c* were parameters measured at or below the transition temperature, and were 0.1 and 1 s^−1^, respectively.

**Figure 3:**
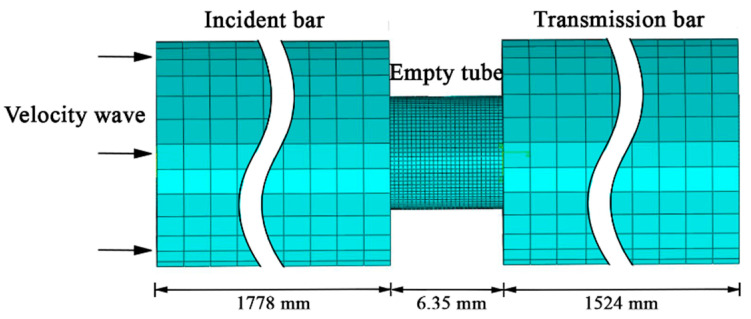
Finite element model of empty tube under split Hopkinson pressure bar.

Dynamic Explicit in Abaqus (Version 2018, Dassault Systems Inc., Paris, France) was selected as the solver. The property of contact was set as hard contact, ignoring friction interaction for all the pairs of surfaces. Part of the finite element model of the filled tube under a split Hopkinson pressure bar is shown in Figure 3. The middle parts of the transmission bar and the incident bar are omitted due to the lengths of these two parts. The velocity waveform, which has been directly loaded on the end-face node of the incident bar near the impact bar, should have twice the waveform length of the impact bar, which was 914.4 mm according to the theory of a one-dimensional elastic wave; meanwhile, the maximum velocity of the loaded waveform should be half of the impact velocity, which was 6150 mm/s. The step time for the Dynamic Explicit was set as 0.8 ms, so as to let the stress wave be transmitted completely. The responses were recorded every 1% of the time node.

**Figure 4:**
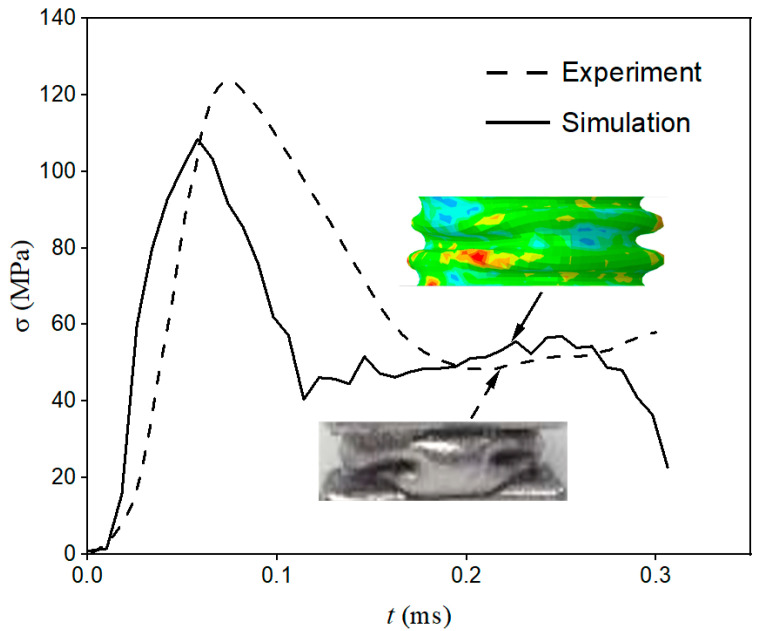
Stress–time curve of empty tube experiment and simulation.

After the explicit nonlinear dynamic analysis was performed, field output was selected to trace the axial strain–time relationship on the typical elements of the transmission bar. Thus, the nominal stress–time curve can be drawn using Formula (3) as shown in Figure 4. Meanwhile, the axial-displacement–time relationship of two ends of the tube was captured, and therefore the nominal strain–time curve could be acquired. Consequently, the nominal stress–strain curve can be obtained by eliminating the time variable.

The simulated post-buckling shape is shown in Figure 4, where the red area indicates the maximum stress. The dangerous stress area is at the protruding part, which is consistent with our common sense. Comparing the experimental and simulated post-buckling shape diagrams and stress–time curves in Figure 4, it can be found that the simulation could be satisfactory for the simulation of the crushing process.

### 3.2. Modeling of Tube Filled with Nanofluidic System

**Figure 5:**
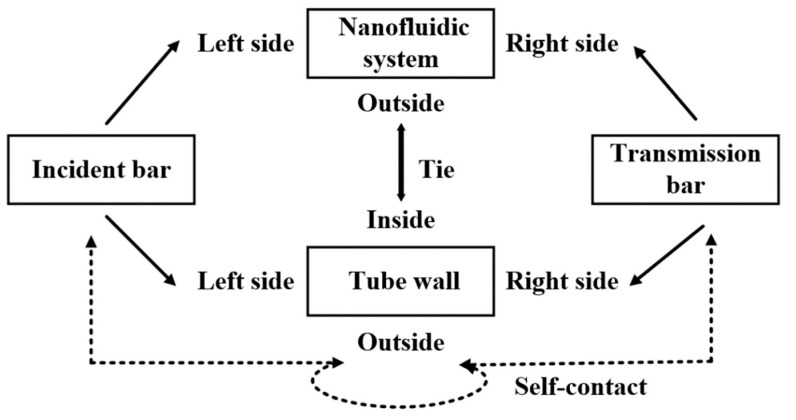
Setting of constraints and contacts.

**Figure 6:**
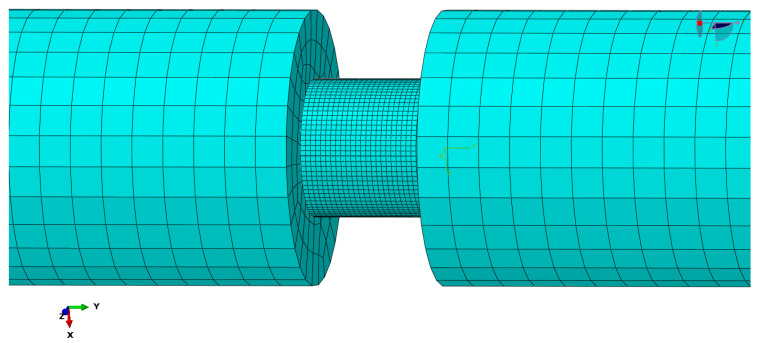
Mesh generation of assembly.

The model and the solution process were verified for a nanofluidic-system-filled tube. This filled tube has a filling nanofluidic system inside, unlike the empty tube. The geometric settings were the same as those of empty one, and the constraints and contacts between each component are shown in Figure 5. The mesh generation is shown in Figure 6.

The infiltration process of the nanofluidic system takes place at the nano scale, which cannot be accurately described by numerical simulations on the macro scale. Notably, the main mechanical characteristic of the nanofluidic system considered here is compressibility, represented by the volumetric change in the system. To this end, the process of the infiltration of the liquid molecules into pores can be mapped to a porous media compaction. The constitutive model of porous media can be adopted to describe the volume response of the nanofluidic system, where the nanofluidic system is equivalent to a porous media material.

The Mie–Hugoniot equation of state was adopted to describe the compressibility of the nanofluidic system. The common form is:

(8) $$p=\Gamma_{0}\rho_{0}E_{m}+p_{H}(1-\frac{\Gamma_{0}\eta }{2})$$

**Figure 7:**
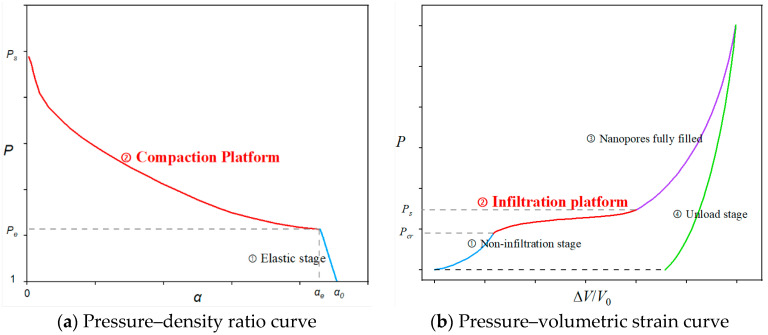
Mapping of nanofluidic system and porous media.

In this expression, *P*_H_ is the Hugoniot pressure, which is only a function of density; *η* is the nominal volumetric compression strain which equals 1 − *ρ*_0_/*ρ*_s_; ρ_0_ is the current mass density; and *E*_m_ is the specific energy, equal to the sum of the work done by the stress and the heat-generation rate. Ulteriorly, the *P*-*α* equation of state, which is designed for the compaction of the porous media, was performed to illustrate the crashing progress shown in Figure 7a, which can be expressed by the following formula:

(9) $$P(\alpha )=-(p_{s}-p_{e}){\left(\frac{\alpha -1}{\alpha_{e}-1}\right)}^{0.5}+p_{s}$$

For the porous media, *P*_*s*_ corresponds to the crushing pressure. *P*_*e*_ corresponds to the compaction pressure, which can be mapped to the infiltration pressure *P*_cr_ of the nanofluidic system. *α* is the density ratio, representing the compaction density to the current density. For fully compacted materials, *α* = 1; otherwise, *α* > 1. Therefore, *α* can be expressed in the form of porosity *n* as:

(10) $$\alpha =\frac{\rho_{s}}{\rho }=\frac{V}{V-V_{p}}=\frac{1}{1-\frac{V_{p}}{V}}=\frac{1}{1-n}$$

In this formula, *V* and *V*_*P*_ are the current volume and initial void volume of the porous media. The infiltration of the nanofluidic system and the compaction process of the porous media (the red curves in Figure 7a,b) showed their corresponding relationship in different coordinate axes.

For the modeling validation, the split Hopkinson pressure bar test on a Fluka 100-C_8_ silicone gel nanofluidic-system-filled tube was simulated and discussed. The speed of sound in the water medium is 1,497,000 mm/s, both slope *s* and Gamma_0_ are equal to 0, and the porosity and the infiltration pressure, which are shown in Table 1, were measured to be 0.26 and 16 MPa, respectively.

Similarly, the nominal stress–strain curve shown with a solid line in Figure 7 can be obtained through the abovementioned method, which was comparable with the experimental one (dashed line). The simulated post-buckling shape is also shown in Figure 4. The red area indicates the dangerous stress area, which is at the protruding part. It proves the correctness of post-buckling shape simulation. However, it is obvious that compared with the simulation curve, the experimental curve was relatively lagging on the time axis, which was reflected in the later appearance of the initial buckling peak stress and the later stress increase in the nanofluidic infiltration stage. The main reasons for this were: First, the defect of the end face of the experimental specimen caused the increase in compression stress in the initial buckling stage to be relatively slow, and its time curve had a certain slope, while the initial buckling of the finite element model and the crushing stress increased instantly. This difference caused the overall experimental curve to lag by about 70 μs. Second, the finite element simplifies the split Hopkinson pressure bar to a uniform crushing process, ignoring the decrease in the speed of the incident bar during the impact, which causes the experimental curve to lag behind the simulated curve in the later crushing period. However, due to the relatively large incident impact energy, this effect is not very significant. 

**Figure 8:**
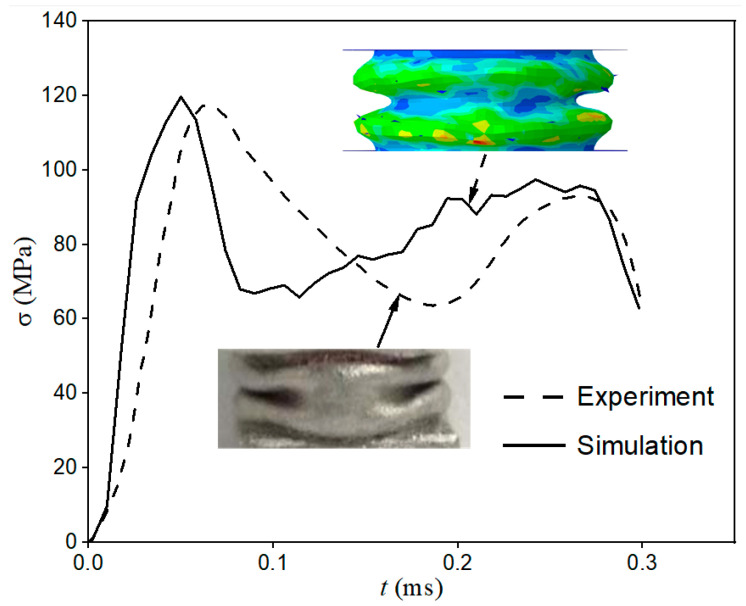
Stress-time curve of Fluka 100-C_8_ silicone gel experiment and simulation.

Despite the existence of the abovementioned hysteresis effect, the finite element results are in good agreement with the experimental results on the whole. Moreover, simulated post-buckling shape diagrams in Figure 8 also indicate that the simulation is satisfactory for the crushing process, and can accurately reflect the influence of nanofluid on the mechanical properties of filled tubes.

### 3.3. Application of Potential Nanofluidic-System-Filled Tube

The above verified modeling methodology has been applied to a tube filled with silica-aerogel functionalized liquid to explore its potential application. A silica-aerogel functionalized liquid with different solid/liquid ratios R_M_ of 1:10, 1:20, and 1:40 was selected. The infiltration and compaction pressure were measured as 5 MPa and 15 MPa, respectively, from quasi-static compression experiment as shown in Table 2, which will not be involved in this paper. The nominal porosity, which depends on the solid/liquid ratio and the material property, was calculated to be 0.49, 0.24, and 0.12, respectively, and other parameters including the filled tube wall were the same as specified in the section above. 

**Figure 9:**
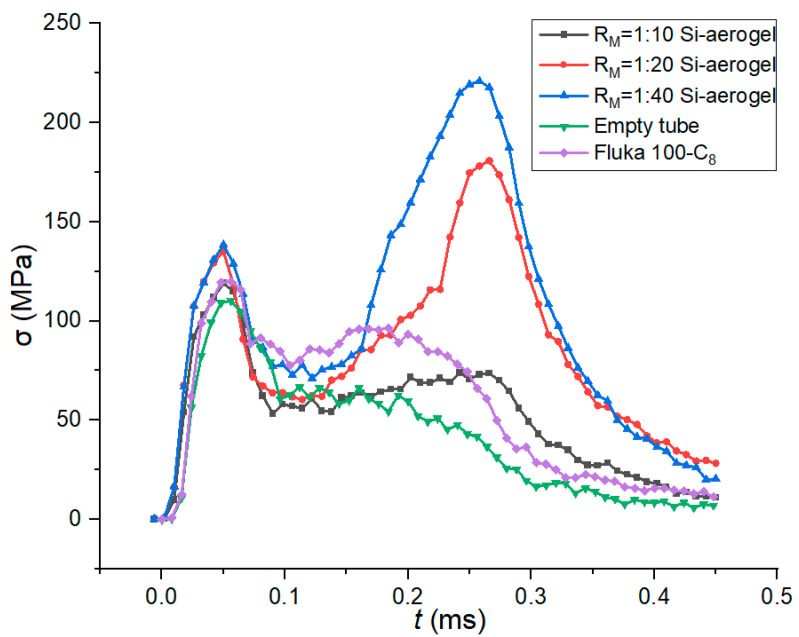
Stress–time curves with different solid/liquid ratios.

Similarly, an explicit algorithm was used for the crushing simulation and the results of the finite element model were computed. The split Hopkinson pressure bar nominal stress–time curves of the silica aerogel filled tube with different solid/liquid ratios are shown in Figure 9. 

**Figure 10:**
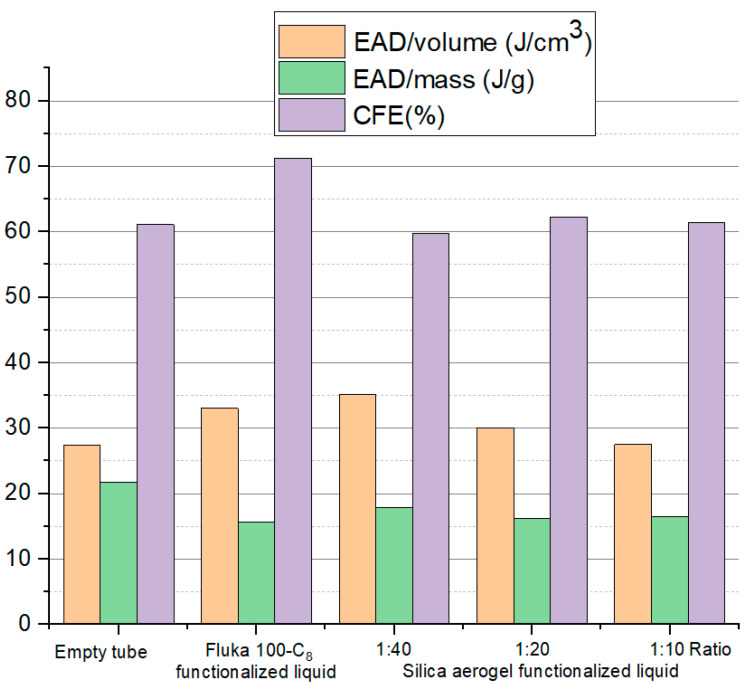
Simulated energy-absorption capabilities of different solid/liquid ratios.

In order to study the energy-absorption capabilities of different filled tubes, three indexes were compared between the tubes as shown in Figure 10, named the energy-absorption density of unit mass, energy-absorption density of unit volume, and crushing force efficiency, which was defined in Section 2.2. The stress wavelength shown in Figure 9 was taken into calculation.

Compared with tubes filled with Fluka 100-C_8_ silicone gel and R_M_ = 1:10, the crush stress increased quickly for silica aerogel with R_M_ = 1:20 at 0.25 ms and R_M_ = 1:40 at 0.17 ms, respectively, which implied a termination of the effective crushing stroke. This represents that all the nanopores of the nanofluidic system were filled at this time.

Figure 10 shows that, except for the EAD of unit volume, the energy-absorption capability indexes of the filled tube were even worse than the empty tube, indicating that the silica gel functionalized liquid was inefficient in improving energy absorption. The reason for could be that the infiltration pressure was as low as 5 MPa, leading to the premature and complete filling of the nanopores, which was not conducive to suppressing the impact response.

With the consideration of the dissatisfying properties of the abovementioned silica aerogel, aerogels with a more appropriate size of pore, which leads to a greater infiltration pressure, are our perfect choice, and silica aerogel purchased from Kemike Inc. suited the purpose. The properties are summarized as follows: Pore size between 10 and 15 nm, density 0.11 g/cm^3^, particle size 10 μm, specific surface area 920 m^2^/g, micro-pore volume 1700 mm^3^/g, thermal stability 900 °C, and hydrophobic angle 145° [17]. Moreover, the added electrolyte could increase the infiltration pressure of the system according to a previous study [18,19]. Therefore, silica-aerogel functionalized NaCl solution liquids with different salinities (5%, 12.5%, and 20%) of the same mass/liquid ratio R_M_ = 1:20 were chosen as the filling material, and the quasi-static compression experiments were conducted where the initial infiltration pressure and compaction pressure [17] were measured as shown in Table 3.

The reason for adding NaCl to increase the infiltration pressure of the nanofluidic system is that Na^+^ is a structurally generated ion that can aggregate surrounding water molecules and guide their hydrogen atoms to adjacent water molecules, thus effectively enhancing the hydrogen-bonding interaction between molecules [20,21]. This microscopic effect enhances the liquid macroscopic surface tension, resulting in an increase in the infiltration pressure.

However, the higher infiltration pressure gave the liquid in the pores a higher potential energy state, in which was easier for water molecules to seep out of the pores when the applied pressure was removed, resulting in a reduction in residual strain. In addition, the repulsion of the nanoporous material tube and the charged particles expanded the gap between the solid/liquid interface of the nanopores [22], resulting in a reduction in the available pore volume, especially for large-specific-surface-area materials such as aerogel. Although the increase in infiltration pressure promoted growth of the energy-absorption density, the reduction in the residual strain and the reduction in the effective pore volume had the opposite effect; consequently, the final energy-absorption density was the result of competition between the two.

**Figure 11:**
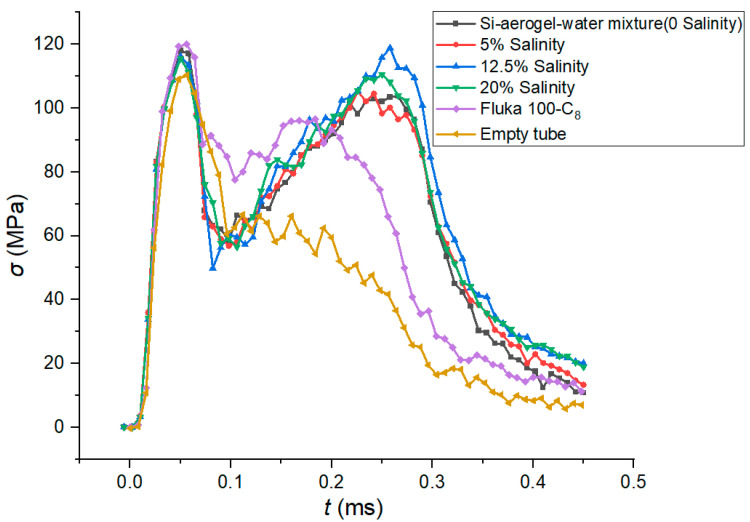
Stress–time curves with different salinities.

The crush simulation results of the silica-aerogel-filled tubes with different salinities are shown in Figure 11. Comparing the stress–strain curves of the empty, silica-aerogel-functionalized-liquid-filled, and Fluka-100-C_8_-silicone-gel-filled tubes, it can be found that the curve of the silica-aerogel functionalized liquid is flatter and higher than other two. The peak crushing stress of the silica-aerogel functionalized liquid is closer to its mean crushing stress, whereas the crushing stress increases significantly when the impact time reaches 0.15 ms, compared to that of the Fluka 100-C_8_ silicone gel. This flatness of the silica-aerogel functionalized liquid is due to its high porosity, which results in high infiltration pressure within the observed crushing stroke.

**Figure 12:**
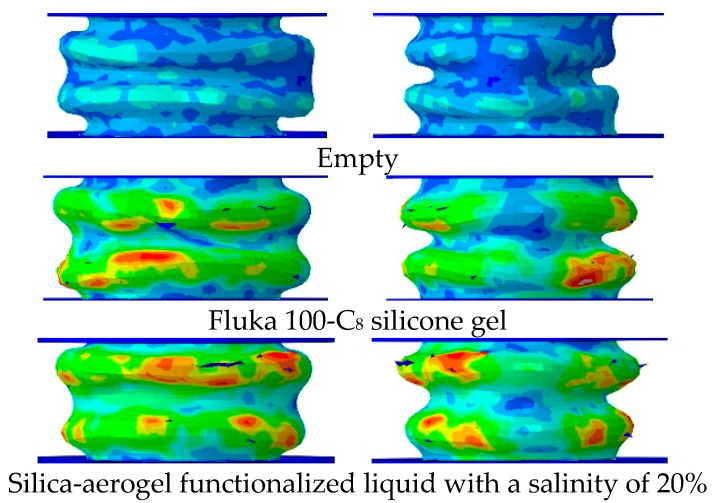
Buckling shapes after crush process.

Figure 12 gives the buckling shapes of the silica-aerogel functionalized liquid with a salinity of 20%, which is compared with those of the Fluka 100-C_8_ silicone gel and empty tube. The overall shape and change trends were similar. Among them, the empty tube is near to free buckling without restraint inside, and the middle part of it is concave, which is in contrast to those of the filling tubes. Although the middle part of the filled tube wall still has a certain degree of indentation, the more remarkable phenomenon is the outward bulging and swelling at the ends. The comparison between both filled tubes intuitively reflects the effects of the internal pressure of the nanofluidic system on the buckling shape. It is obvious that under the internal pressure, the buckling progressed along with the in-plane tension of the tube wall, which is significant and accompanied by high infiltration pressures. Consequently, the concavity in the middle part is weakened and the outward bulging at the end is strengthened.

**Figure 13:**
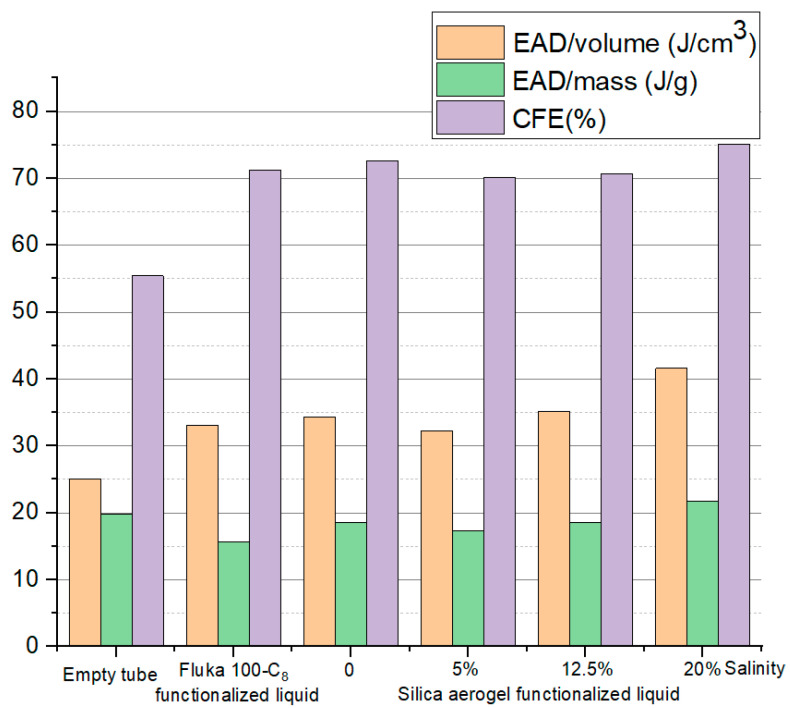
Simulated energy-absorption capabilities with different salinities.

Figure 13 illustrates the calculated energy-absorption capabilities for filled tubes with silica-aerogel functionalized liquid and Fluka 100-C_8_ silicone gel functionalized liquid, as well as the empty tube. It can be concluded that the selected aerogel liquid has significant energy-absorption density and crush-force efficiency compared to the empty tube and the existing Fluka-100-C_8_-silicone-gel-material-filled tube, especially as the crush force efficiency of silica aerogel-liquid filled tube was as high as 70%, which is comparable to the Fluka 100-C_8_ silicone gel. Moreover, it shows that energy-absorption capabilities of the proposed aerogel material can be compatible with Fluka 100-C_8_ silicone gel with a much lower solid/liquid ratio. In short, we found that by adjusting the solid/liquid ratio, nanomaterial type, and ion concentration, the nanofluidic system can achieve the required total pore volume and infiltration pressure, thereby achieving better compressibility and higher crushing stress.

## 4. Conclusions

In this paper, the simulation method for split Hopkinson pressure bar experiment devices and nanofluidic systems was established by the derived two-wave method. The mechanical responses of tubes filled with different systems were obtained. The key factors affecting energy absorption were explored by comparing the stress–time curves and buckling shapes of different systems. Compared with the existing simulation methods, this methodology can reflect the characterization of the microscopic pore infiltration process of nanofluids in the macroscopic volumetric change which reveals the influential mechanism of its molecular-scale solid–liquid infiltration behavior.

The study also reveals that the filling of the nanofluid system only affects the post buckling stage of the filled tube, which was reflected in the increase in the crushing stress and energy-absorption capability and the change in the buckling shape. The simulation illustrated that the solid/liquid ratio determines the effective compression stroke, and the infiltration pressure determines the post-buckling crushing stress of the tube. On this basis, it is valid to consider that the infiltration pressure and the solid/liquid ratio can be independently adjusted to obtain the required effective compression stroke and post-buckling crushing stress level, respectively, which can simultaneously improve the energy-absorption level and crushing force efficiency of the tube structure without raising the buckling force peak.

## Figures and Tables

**Table 1:** Parameter settings of Fluka 100-C_8_ silicone gel nanofluidic system.

Slope *s*	Gamma_0_	Sound Speed (mm/s)	Critical Infiltration Pressure (MPa)	Nominal Porosity
0	0	1,497,000	16	0.26

**Table 2:** Parameter settings of silica-aerogel functionalized liquid with different mass/liquid ratios.

Mass/Liquid Ratio	Critical Infiltration Pressure (MPa)	Compaction Pressure (MPa)	Nominal Porosity
1:10	5	15	0.49
1:20	5	15	0.24
1:40	5	15	0.12

**Table 3:** Silica aerogel material properties.

Salinity	Density (kg/m^3^)	Critical Infiltration Pressure (MPa)	Full Infiltration Pressure (MPa)	Nominal Porosity
20%	791	30	42	0.24
12.5%	762	28	38	0.24
5%	741	25	32	0.24
0	722	21	25	0.24

## Data Availability

The data presented in this study are available on request from the corresponding authors.
